# Utilizing Point-of-Care Ultrasound for Timely Diagnosis of Retained Products of Conception in Resource-Limited Settings

**DOI:** 10.7759/cureus.79776

**Published:** 2025-02-27

**Authors:** Jonathan Schonert, Brandon Wilson, Heather Tucker, Joseph Minardi

**Affiliations:** 1 Emergency Medicine, St. Luke's Hospital - Chesterfield, Chesterfield, USA; 2 Emergency Medicine, West Virginia University School of Medicine, Morgantown, USA; 3 Emergency Medicine, West Virginia University, Morgantown, USA

**Keywords:** bedside ultrasound, incomplete abortion, incomplete miscarriage, miscarriage, pocus (point of care ultrasound), postpartum bleeding, retained products of conception (rpoc)

## Abstract

Vaginal bleeding following dilation and curettage or miscarriage is a common presentation in the emergency department. Diagnosing retained products of conception (RPOC) can be challenging in resource-limited settings, as it often requires transvaginal ultrasonography documented by a certified radiologist. This case report describes a young woman who presented after hours to a critical access emergency department with vaginal bleeding six days post-dilation and curettage for spontaneous abortion. Physical examination findings were nonspecific, and radiology ultrasound was unavailable. Point-of-care ultrasound (POCUS) was performed, revealing echogenic material within the endometrial cavity with internal vascularity on color Doppler, consistent with RPOC. The patient was transferred to a higher-level care facility and underwent a second dilation and curettage. This case underscores the utility of POCUS as a valuable diagnostic tool in emergency settings and highlights sonographic findings characteristic of RPOC.

## Introduction

Vaginal bleeding following dilation and curettage or miscarriage can be a challenging presentation in the emergency department. From an emergency physician’s perspective, particularly during overnight shifts, these cases where bleeding is not excessive often present with subtle or nonspecific findings. While human chorionic gonadotropin (hCG) levels can remain elevated for weeks after such events [1], trending these levels to determine retained products of conception is more valuable in the outpatient follow-up setting rather than in the acute setting. Clinical and laboratory signs of complications such as infection are often nonspecific as well, making ruling out serious diagnoses challenging. Retained products of conception (RPOC) is a critical consideration in such presentations and is typically confirmed through radiology-performed ultrasonography [2]. However, in settings with limited access to imaging, obtaining a radiology-performed ultrasound may be difficult. Transfers to tertiary care centers for further evaluation may encounter resistance from both patients and facilities, particularly in the absence of clear emergent indications. On top of that, delay in diagnosing RPOC can lead to significant complications, including uterine infection, intrauterine adhesions and scarring, infertility, and massive hemorrhage. This underscores the importance of timely and accurate diagnosis in the acute setting [3].

Point-of-care ultrasound (POCUS) is increasingly recognized as a valuable diagnostic tool in emergency medicine, especially in resource-limited settings. Its indications continue to expand, and it offers a practical alternative for evaluating post-partum or post-procedural vaginal bleeding when radiology-performed ultrasonography is unavailable. POCUS can aid in identifying signs of endometritis or RPOC, significantly impacting patient management and outcomes.

This case highlights the utility of POCUS in diagnosing RPOC in a patient presenting to a critical access emergency department after hours with post-procedural vaginal bleeding.

## Case presentation

A young woman presents after hours to a critical access emergency department with vaginal bleeding and pelvic cramps six days after undergoing dilation and curettage for spontaneous abortion. Vital signs were unremarkable. Patient was Rh-positive. Of note, radiology-performed ultrasound was not available, even on an on-call basis at this facility.

**Figure 1:**
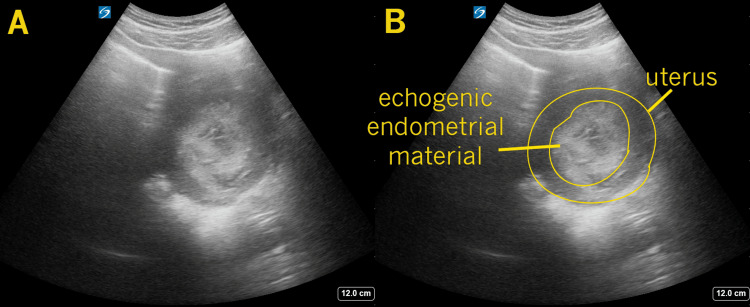
Transverse Uterus In these transverse images of the uterus, obtained via a transabdominal technique, significantly thickened, echogenic material is seen within the endometrial cavity (annotated in frame B). This finding is highly suggestive of retained products of conception.

**Figure 2:**
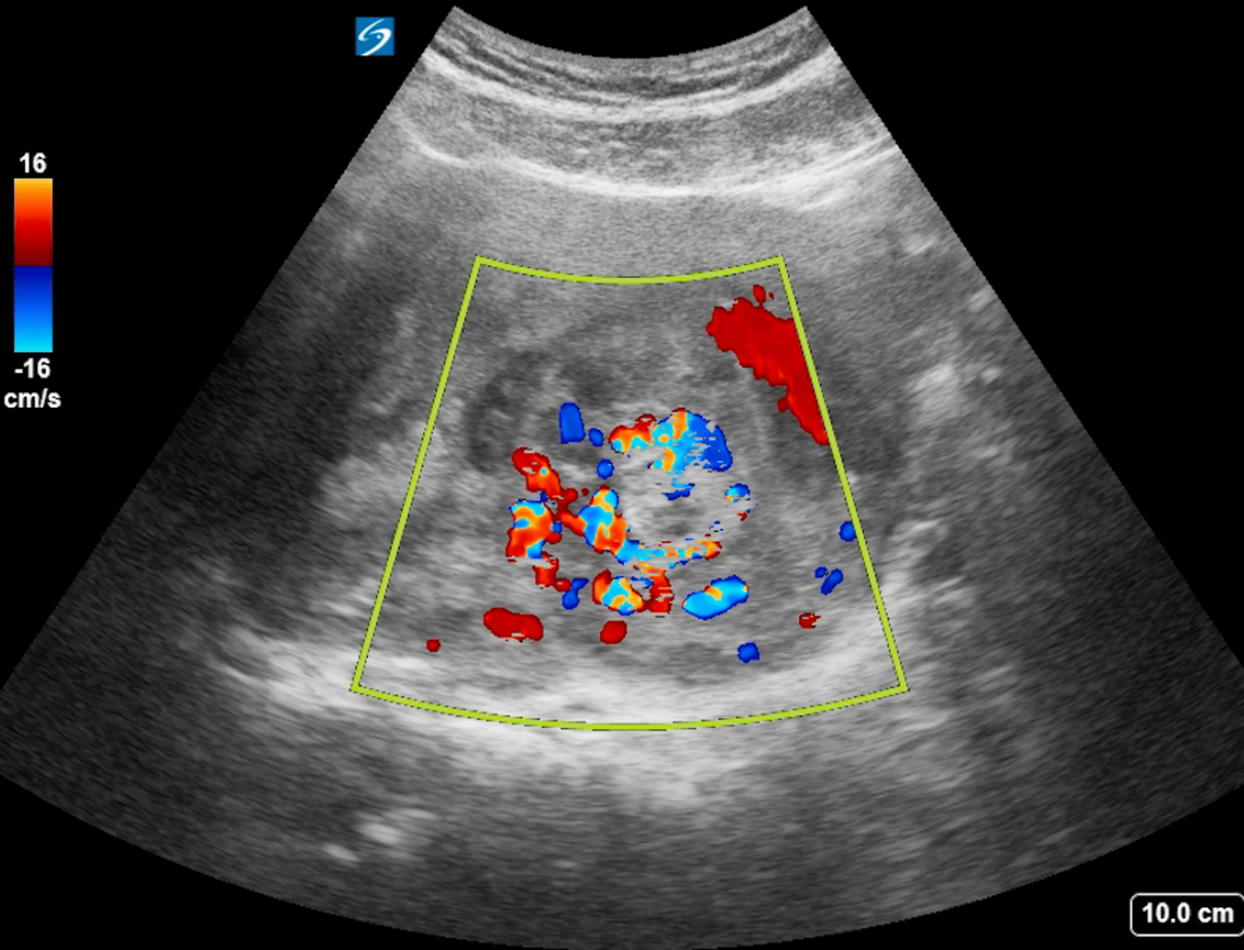
Transverse Uterus Color Doppler In this transverse view of the uterus, obtained via a transabdominal technique, color Doppler is applied to the thickened, echogenic endometrial material, displaying internal vascularity. This finding is highly specific for retained products of conception.

Abdomen exam revealed tenderness with deep palpation to the lower pelvic region, somewhat midline. Pelvic examination revealed a small amount of blood coming from a closed cervical os. Transabdominal point-of-care ultrasound was performed during the initial history and physical examination. Complex echogenic material was seen within the endometrial cavity (as seen in Figure 1) with internal vascularity demonstrated on color Doppler interrogation (as seen in Figure 2). These findings were consistent with retained products of conception.

The patient was transferred to a higher-level care facility with gynecologist specialities for further surgical evaluation and management. IV antibiotics were started and given during transport. The patient required a second dilation and curettage the following day and was discharged shortly after this without complication.

## Discussion

RPOC should be a key consideration in postpartum patients presenting with pain or bleeding, as these symptoms can signal potentially serious complications. The incidence of RPOC varies, occurring in approximately 0.5% to 2% of all abortions [4], including spontaneous, medical, and surgical terminations. Risk factors for RPOC are early postpartum hemorrhage, assisted reproductive technology and previous RPOC [5]. Patients typically present within a few days to a week post-procedure, with bleeding being the hallmark symptom. Pain and fever, while less common, may suggest infection or more advanced complications.

Diagnosing RPOC in the ED can be challenging, particularly in resource-limited or after-hours settings. Physical examination findings, such as an open cervical os or light-to-moderate vaginal bleeding, are nonspecific and cannot definitively rule in or rule out RPOC. Laboratory studies, including complete blood count and serial hCG levels, may provide some context but are rarely diagnostic in isolation. Timely imaging is the primary component in the diagnosis and guides management.

In critical access or rural emergency departments, where access to radiology-performed ultrasound may be limited, POCUS offers a valuable alternative. Typical findings for RPOC that have been studied and published usually pertain to transvaginal ultrasonography. These same features can often be visualized on bedside transabdominal ultrasound as well. Echogenic masses in the endometrial cavity can be a very sensitive finding for RPOC [6]. One of the most specific findings for RPOC on POCUS is endometrial vascularity, which is often seen using color Doppler. Increased vascularity within echogenic material in the endometrial cavity strongly suggests RPOC. Additionally, measuring the endometrial stripe thickness can provide important diagnostic clues, with thickness >7-10 mm being highly predictive of RPOC [7,8].

Performing POCUS for postpartum bleeding involves using a curvilinear transducer in the suprapubic position. The authors recommend initially identifying the bladder as a reliable landmark, then subsequently identifying the uterus in relation. The uterus may be seen posterior and sometimes superior to the bladder depending on the level of bladder distention and relative size of the uterus. Once identified, evaluate the entirety of the uterus from the fundus to the cervical portion. Sweeps in the longitudinal and transverse planes should focus on the endometrial stripe, with careful attention to measuring thickness and evaluating for vascularity using color Doppler imaging [9]. It would be wise to also do sweeps through the peritoneum looking for free fluid and the ovaries inspecting for irregularity or potential ectopic pregnancies. This bedside approach can provide timely, actionable information, helping clinicians determine the necessity for gynecologic consultation, transfer to a higher level of care, or initiation of antibiotics and other interventions.

Failure to diagnose and manage RPOC promptly can result in serious complications, including uterine infection, massive hemorrhage, and long-term reproductive consequences such as intrauterine adhesions and infertility [10]. While POCUS findings alone cannot definitively confirm RPOC, they can significantly increase clinical suspicion and support the decision to transfer or initiate treatment, especially in resource-limited settings. As this case demonstrates, early identification and intervention can lead to favorable outcomes, even in challenging circumstances.

## Conclusions

The integration of POCUS into the evaluation of postpartum or post-procedural vaginal bleeding in emergency settings - particularly in limited-resource or rural environments - can enhance diagnostic accuracy and improve patient outcomes. Recognizing and acting on key ultrasound findings, such as endometrial masses and vascularity, and thickened endometrial stripe, can guide timely and appropriate management of RPOC. This case underscores the importance of POCUS as a valuable tool for emergency physicians and highlights the potential for improved care in critical access settings.
